# Caveolin-1 Promotes Cellular Senescence in Exchange for Blocking Subretinal Fibrosis in Age-Related Macular Degeneration

**DOI:** 10.1167/iovs.61.11.21

**Published:** 2020-09-14

**Authors:** Hideyuki Shimizu, Kazuhisa Yamada, Ayana Suzumura, Keiko Kataoka, Kei Takayama, Masataka Sugimoto, Hiroko Terasaki, Hiroki Kaneko

**Affiliations:** 1 Department of Ophthalmology, Nagoya University Graduate School of Medicine, Nagoya, Japan; 2 Department of Ophthalmology, National Defense Medical College, Japan; 3 Department of Mechanism of Aging, National Center for Geriatrics and Gerontology, Obu, Aichi, Japan; 4 Institutes of Innovation for Future Society, Nagoya University, Nagoya, Japan

**Keywords:** caveolin-1, age-related macular degeneration, subretinal fibrosis, epithelial–mesenchymal transition, cellular senescence

## Abstract

**Purpose:**

To determine whether caveolin-1 (i) prevents epithelial–mesenchymal transition in the RPE and laser-induced subretinal fibrosis and (ii) promotes or inhibits cellular senescence in the RPE.

**Methods:**

We examined laser-induced subretinal fibrosis and RPE cell contraction in wild-type and *Caveolin-1* knockout (*Cav-1^−/−^*) mice treated with or without cavtratin, a cell-permeable peptide of caveolin-1. The senescence marker p16^INK4a^ was measured in RPE tissues from patients with geographic atrophy and aged mice, laser-induced subretinal fibrosis, and primary human RPE cells. Human RPE was examined by TUNEL staining, reactive oxygen species generation, cell viability, and senescence-associated β-galactosidase staining.

**Results:**

The volume of subretinal fibrosis was significantly smaller in cavtratin-injected eyes from wild-type mice than in control eyes from wild-type, *P* = 0.0062, and *Cav-1^−/−^* mice, *P* = 0.0095. Cavtratin treatment produced significant improvements in primary RPE cell contraction in wild-type, *P* = 0.04, and *Cav-1^−/−^* mice, *P* = 0.01. *p16^INK4a^* expression in the RPE was higher in patients with than without geographic atrophy. *p16^INK4a^* was expressed in 18-month-old but not 2-month-old wild-type mouse eyes. p16^INK4a^ and collagen type I antibodies showed co-localization in subretinal fibrosis. Cavtratin did not affect RPE cell apoptosis or reactive oxygen species generation, but decreased cell viability and increased senescence-associated β-galactosidase–positive cells.

**Conclusions:**

Enhanced expression of caveolin-1 successfully blocked epithelial–mesenchymal transition of RPE and the reduction of subretinal fibrosis in mice. Nevertheless, in exchange for blocking subretinal fibrosis, caveolin-1 promotes RPE cellular senescence and might affect the progression of geographic atrophy in AMD.

AMD is a leading cause of blindness in most industrialized nations.^1^^,^^2^ Neovascular AMD, one of the two different forms of AMD, is characterized by invasion of choroidal neovascularization (CNV) into the sensory retina.^3^ Advanced CNV growth leads to subretinal fluid accumulation or, at worst, subretinal hemorrhage with severe visual damage.^4^ Enhanced expression of the proangiogenic cytokine VEGF has been confirmed in patients with neovascular AMD, and anti-VEGF drugs are currently the standard treatment for neovascular AMD.^5^^–^^7^ However, despite repeated administration of anti-VEGF drugs, CNV growth in some patients is not successfully suppressed by these drugs. In these patients, CNV progresses to subretinal fibrosis, an end-stage condition characterized by the formation of a fibrous plaque and disciform scar between the sensory retina and RPE.^8^^,^^9^ Previous studies have demonstrated the biological involvement of epithelial–mesenchymal transition (EMT) in RPE cells in patients with subretinal fibrosis secondary to neovascular AMD^10^: RPE cells transform into fibrotic cells, then migrate and spread beneath the sensory retina. The visual acuity of patients with advanced neovascular AMD is extremely poor, and the presence of subretinal fibrosis is reportedly a risk factor for reduced visual function in patients with neovascular AMD.^11^

Caveolin-1 is a 21- to 24-kDa integral membrane protein that has been extensively investigated in many biochemical studies. Caveolin-1 is present at the endoplasmic reticulum and Golgi complex and predominantly at the plasma membrane. It has also been found in a variety of cells, such as adipocytes, endothelial cells, epithelial cells, and even the lens epithelium in the eye.^12^ Recent studies have shown that caveolin-1 plays a key biological role in cancer-related processes, including tumor metastasis and angiogenesis.^13^^–^^15^ Moreover, Jiang et al.^16^ revealed that cavtratin, a cell-permeable peptide of caveolin-1, inhibits CNV growth and migration of microglia/macrophages via JNK in mouse neovascular AMD models. Caveolin-1 also plays an important role in the EMT of cancer biology and tissue fibrosis.^13^^,^^15^^,^^17^^,^^18^ Similarly, we showed that caveolin-1 plays a pivotal role in the pathogenesis of proliferative vitreoretinopathy, in which floating RPE cells in the vitreous of eyes with retinal detachment attach to the surface of the retina, resulting in the initiation of EMT and migration of the RPE cells as fibrotic cells.^19^ In our previous study, *Caveolin-1* knock-down and knock-out enhanced EMT in both the human and mouse RPE, whereas enhanced expression of caveolin-1 blocked EMT.

Geographic atrophy (GA), the other form of AMD, is characterized by atrophy of the RPE. No treatment that can halt or reverse the progression of GA is currently available. Various risk factors are thought to be responsible for the pathogenesis of GA; the accumulation of *Alu* RNA caused by *DICER1* deficiency in the RPE is reportedly capable of triggering the activation of NLRP3 inflammasomes.^20^^,^^21^ In our recent study, *Alu* RNA induced the upregulation of *p16^INK4a^*,^22^ one of the key elements in human cellular senescence.^23^ Interestingly, the overexpression of caveolin-1 reportedly arrests mouse embryonic fibroblasts in the G0/G1 phase of the cell cycle and promotes cellular senescence.^24^ In addition, cellular senescence is thought to be an important key underlying the development of GA in patients with AMD.^25^^–^^27^

Therefore, in the current study, we sought to understand whether caveolin-1 (i) plays a role in the prevention of subretinal fibrosis in neovascular AMD and (ii) promotes or inhibits cellular senescence, which can affect the progression of GA in exchange for blocking EMT in RPE cells.

## Methods

### Mouse Model of Subretinal Fibrosis After Laser-Induced CNV

Male wild-type C57BL/6J mice (CLEA, Tokyo, Japan) or *Caveolin-1* knock-out (*Cav-1^−/−^*) mice aged 6 to 8 weeks were used.^19^ For all procedures, the animals were anesthetized with ketamine (133 mg/kg body weight) and xylazine (5.3 mg/kg body weight) with additional dosing if necessary, and the pupils were dilated with a combination of 0.5% tropicamide and 0.5% phenylephrine (Mydrin-P; Santen, Osaka, Japan). Subretinal fibrosis following laser-induced CNV was generated as previously described.^28^ Briefly, four laser photocoagulation spots (532-nm laser; power, 180 mW; duration, 100 ms; diameter, 75 mm) (Novus Verdi; Coherent Inc., Santa Clara, CA) were placed in the fundus of each eye on day 0 by an individual blinded to the group assignments. The laser spots were created around the optic nerve using a slit-lamp delivery system, and a coverslip was used as a contact lens. The morphologic end point of the laser injury was the appearance of a cavitation bubble, which is the sign of Bruch's membrane disruption. After completion of laser-induced CNV, cavtratin (500 ng in 1 µL) or antennapedia (1 µL, control) was intravitreally injected. The use of animals in the experimental protocol was approved by the Nagoya University Animal Care Committee. All animal experiments were performed in accordance with the guidelines of the ARVO Statement for the Use of Animals in Ophthalmic and Vision Research.

### Subretinal Fibrosis Volume Measurement and p16 Immunostaining of RPE Flatmounts

The subretinal fibrotic tissue volume and p16 immunostaining were examined in RPE flatmounts 35 days after laser photocoagulation. After euthanasia, the mouse eye cups were fixed in 4% paraformaldehyde and then permeabilized in 1% Triton X-100 (Katayama Chemical, Osaka, Japan) overnight. An RPE flatmount was generated and stained with anticollagen type I antibody (Rockland Immunochemicals Inc., Limerick, PA) or anti-p16 antibody (kindly gifted by the Spanish National Cancer Research Center [Madrid, Spain]) at 4°C overnight followed by Alexa Fluor 488- or 594-tagged secondary IgG secondary antibody (Thermo Fisher Scientific, Waltham, MA). Subretinal fibrosis was visualized using an argon laser (488-nm wavelength). Horizontal optical sections were obtained at 1-mm intervals from the top of the subretinal fibrosis to the surface of the RPE using a confocal laser scanning microscope (Eclipse C1 confocal; Nikon, Tokyo, Japan). The collagen type I–positive subretinal fibrosis images of each layer were stored digitally and measured using ImageJ software (National Institutes of Health, Bethesda, MD). The summation of the whole fluorescent area in each horizontal section was used as an index for the volume of subretinal fibrosis.

### *p16^INK4a^* RT-PCR

Human RPE tissues were obtained from donor eyes from the Minnesota Lions Eye Bank (St. Paul, MN) as previously described.^29^ Genetically wild-type mouse ocular tissues obtained from a breeding colony for ARF-DTR transgenic mice^30^ and cultured human RPE cells (Lonza, Walkersville, MD) were used for the study. The total RNA was reverse-transcribed using a Transcriptor Universal cDNA Master Kit (Roche Diagnostics, Basel, Switzerland), starting with 2  µg of total RNA from each sample. RT-PCR was performed using the Thunderbird Probe qPCR Mix (Toyobo Life Science, Osaka, Japan). RT-PCR cycles with SYBR green consisted of pre-denaturation at 98°C for 2 minutes followed by 45 cycles of denaturing at 98°C for 10 seconds, annealing at 55°C for 10 seconds, and extending at 68°C for 30 seconds. The relative expressions of the target genes were determined using the 2^−△△Ct^ method. Primer sequences are listed in Table. Because the control sample from 2-month-old mice did not show abundant *p16^INK4a^* expression, quantitative RT-PCR was not thought to have been correctly evaluated. Therefore, the PCR products were additionally run on a 1.5% agarose gel with ethidium bromide (10 µg/mL; Sigma-Aldrich, St. Louis, MO), and DNA bands were visualized with UV light.

**Table.** Primer Sequences Used in This Study

Species	Gene Symbol	Forward Sequence (5ʹ-3ʹ)	Reverse Sequence (3ʹ-5ʹ)
Human	p16^INK4a^	GGGGGCACCAGAGGCAGT	GGTTGTGGCGGGGGCAGTT
	GAPDH	GGAAGGTGAAGGTCGGAGTCA	GTCATTGATGGCAACAATATCCACT
Mouse	p16^INK4a^	CCCAACGCCCCGAACT	GCAGAAGAGCTGCTACGTGAA
	18S	AGTCCCTGCCCTTTGTACACA	GATCCGAGGGCCTCACTAAAC

### Cell Culture Assays

Primary human RPE cells (Lonza) were maintained in Dulbecco's modified Eagle's medium and Ham's F12 medium (Sigma-Aldrich) containing 10% fetal bovine serum, penicillin, and streptomycin (Sigma-Aldrich). The primary human RPE cells were transfected with Stealth small interfering RNA (Stealth siRNA; Invitrogen, Carlsbad, CA) targeting *CAVEOLIN-1* (siRNA_*CAV-1*, HSS141466) and the negative control (siRNA_Ctrl).^19^ Before cell culture assays, the RPE cells were treated for 48 hours with or without 2 µM cavtratin. Cell viability was determined using a Cell Proliferation Kit 1 (Roche Diagnostics) according to the manufacturer's instructions. To detect TUNEL-positive apoptotic cells, culture RPE cells were stained with the in situ cell death detection kit (Roche Diagnostics). To detect senescent cells, human RPE cells were fixed and stained with SPiDER-βGal (Dojindo Laboratories, Kumamoto, Japan), which can emit strong and stable fluorescence after the reaction with senescence-associated β-galactosidase. To measure reactive oxygen species generation in human RPE cells, the RPE cells were loaded with 10 µmol/L 5-(and-6)-chloromethyl-2’,7’-dichlorodihydrofluorescein diacetate acetyl ester (CM-H2DCFDA; Molecular Probes, Eugene, OR). We observed the stained cells using a BioImaging Navigator fluorescence microscope (BZ-9000; Keyence, Osaka, Japan).

### RPE Cell Contraction Assays In Vitro

Mouse primary RPE cells were cultured as previously described^19^ and used for the cell contraction assays with a CytoSelect 48-Well Cell Contraction Assay Kit (Cell Biolabs, San Diego, CA) according to the manufacturer's protocol. Briefly, primary wild-type and *Cav-1^−/−^* RPE cells were prepared in each cell contraction matrix with or without 2 µM cavtratin in the culture medium and then added to the top of each collagen gel lattice. Only culture medium was added to another collagen gel lattice as a control. An image of each collagen gel was captured at 48 hours, and the area of each gel was measured using ImageJ software. The area of collagen gel containing no cells was used at 100% for normalization.

### Statistical Analysis

Data are expressed as mean ± standard error (*n* = number of samples). For the in vivo study, we compared parameters between the subretinal fibrotic tissue volume with and without cavtratin using the Mann–Whitney U test. For the in vitro study, values in the control group are expressed as 100% and the parameters were statistically evaluated using the Student *t* test. *P* values of less than 0.05 were considered statistically significant in all analyses.

## Results

### Role of Caveolin-1 in Subretinal Fibrosis Growth In Vivo

**Figure 1.**
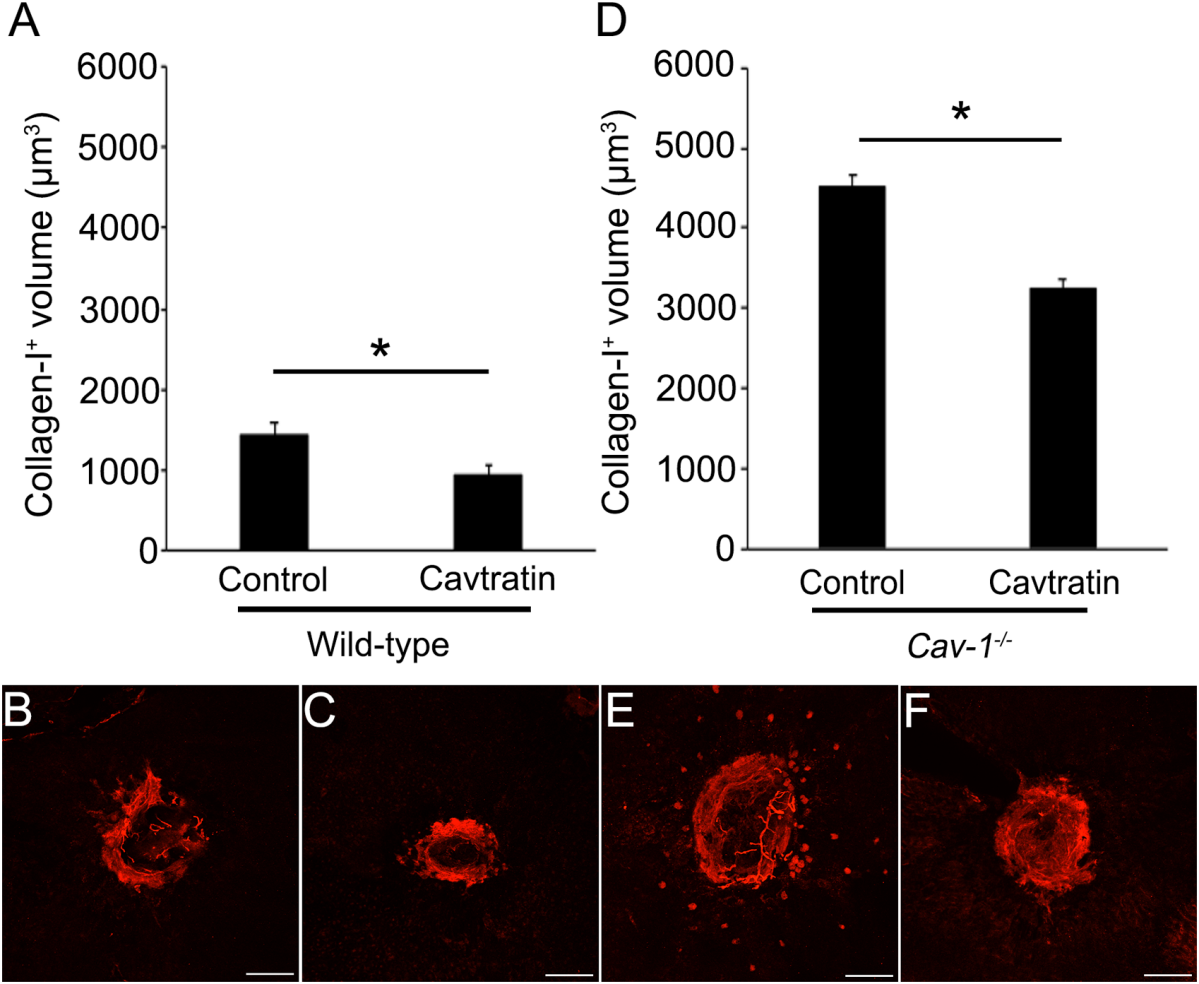
Volume of collagen type I-positive subretinal fibrotic tissue in mice. (**A**) Eyes were collected at day 35 after laser induction. The volume of collagen type I–positive subretinal fibrotic tissue in cavtratin-injected eyes from wild-type mice was significantly smaller than that in sham-injected eyes, *P* = 0.0062. (**B**, **C**) Representative images of collagen type I–positive subretinal fibrotic tissues from wild-type mice. (**D**) The volume of collagen type I-positive subretinal fibrotic tissue in cavtratin-injected eyes from caveolin-1 knockout (*Cav-1^−^^/^^−^*) mice was significantly smaller than that in sham-injected eyes, *P* = 0.0095. (**E**, **F**) Representative images of collagen type I-positive subretinal fibrotic tissues from *Cav-1^−^^/^^−^* mice. ^*^P < 0.05. Scale bars = 100 µm.

First, we examined whether cavtratin, a cell-permeable peptide derived from caveolin-1, decreases the volume of collagen type I-positive subretinal fibrosis in mice (Fig. 1). The volume of collagen type I–positive subretinal fibrosis in cavtratin-injected eyes from wild-type mice, 950.4 ± 116.2 µm^3^, *n* = 25, was significantly smaller than that in control eyes, 1420.4 ± 164.7 µm^3^, *n* = 27, *P* = 0.0062. Similarly, the volume of collagen type I–positive subretinal fibrosis in cavtratin-injected eyes from *Cav-1^−/−^* mice, 3232.5 ± 375.5 µm^3^, *n* = 27, was significantly smaller than that in control eyes, 4502.8 ± 310.6 µm^3^, *n* = 22, *P* = 0.0095. These results indicate that genetic depletion of *Caveolin-1* resulted in an increase in subretinal fibrosis, but that cavtratin functioned in the decrease of subretinal fibrosis growth in both wild-type and *Cav-1^−/−^* mice.

### Role of Caveolin-1 in Mouse RPE Cell Contraction In Vitro

**Figure 2.**
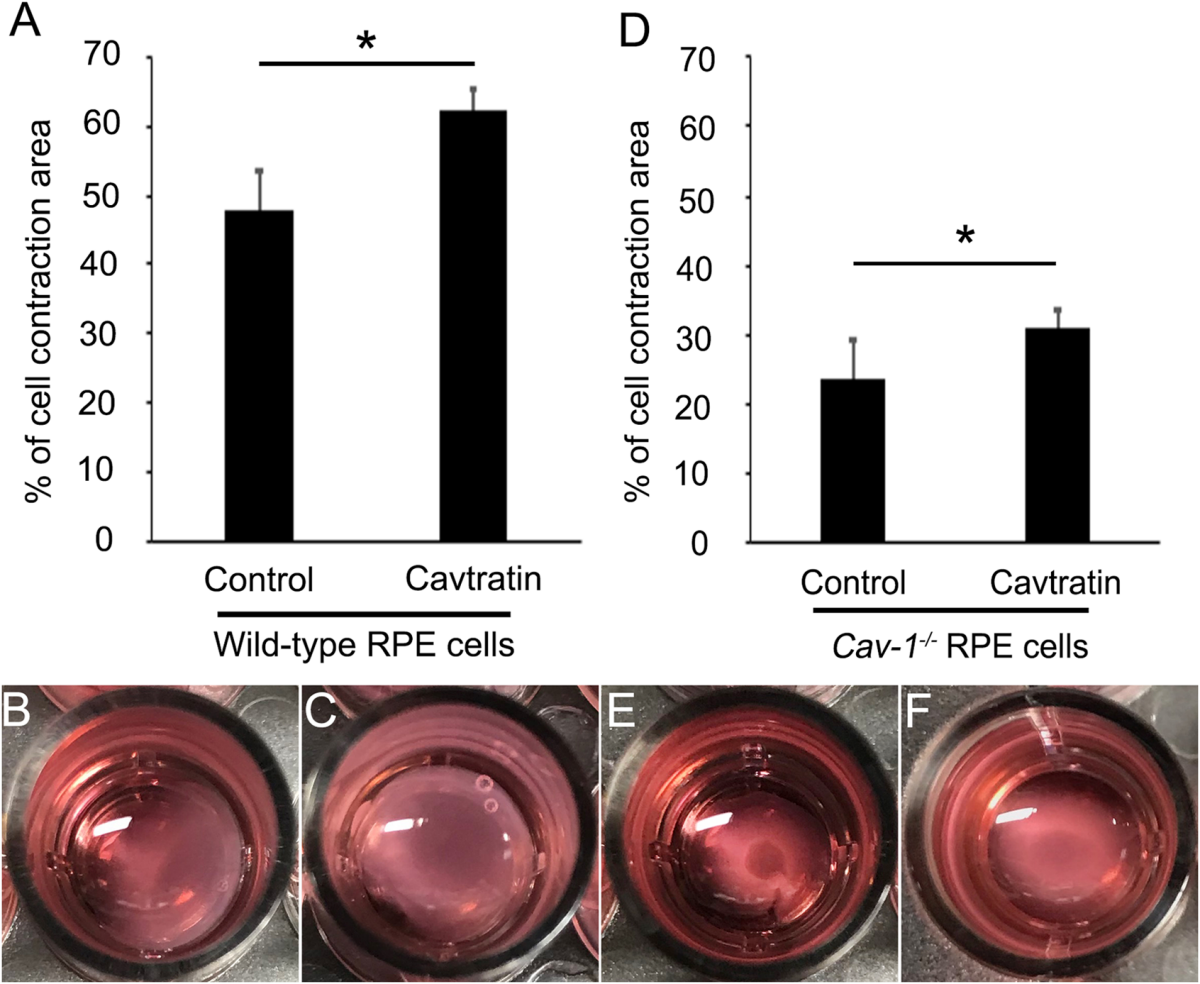
Change in primary mouse RPE cell contraction and caveolin-1. (**A**) The area of contracted RPE cells from wild-type mice with cavtratin treatment was significantly larger than that from controls, *P* = 0.04. (**B**, **C**) Representative images of cell contraction assay from primary RPE cells of wild-type mice. (**D**) The area of contracted RPE cells from Caveolin-1 knockout (*Cav-1^−/−^*) mice with cavtratin treatment was significantly larger than that from controls, *P* = 0.01. (**E**, **F**) Representative images of cell contraction assay from primary RPE cells of *Cav-1^−/−^* mice. ^*^P < 0.05.

We previously demonstrated that caveolin-1 suppresses EMT of RPE cells by migration and scratch assays in vitro.^19^ In the present study, we further examined whether cavtratin blocks human and mouse RPE cell fibrosis in vitro using a cell contraction assay (Fig. 2). The percent of cell contraction in cavtratin-treated primary wild-type mouse RPE cells was 62.5% ± 2.8% (*n* = 6), whereas that in control primary wild-type RPE cells was 48.0% ± 5.4% (*n* = 6). There was a significant difference in wild-type mouse RPE cell contraction depending on cavtratin treatment, *P* = 0.04. Similarly, the percent of contraction in cavtratin-treated primary *Cav-1^−/−^* mouse RPE cells was 30.1% ± 5.6% (*n* = 6), whereas that in control *Cav-1^−/−^* mouse RPE cells was 23.4% ± 6.1% (*n* = 6). There was a significant difference in *Cav-1^−/−^* mouse RPE cell contraction depending on cavtratin treatment, *P* = 0.01. These results indicate that genetic depletion of *Caveolin-1* resulted in the enhancement of RPE cell contraction but that cavtratin functioned in the decrease of RPE cell contraction in both wild-type and *Cav-1^−/−^* mice.

### *p16^INK4a^* Expression in Human RPE and in Retina, RPE, and Sclera of Old Mice

**Figure 3.**
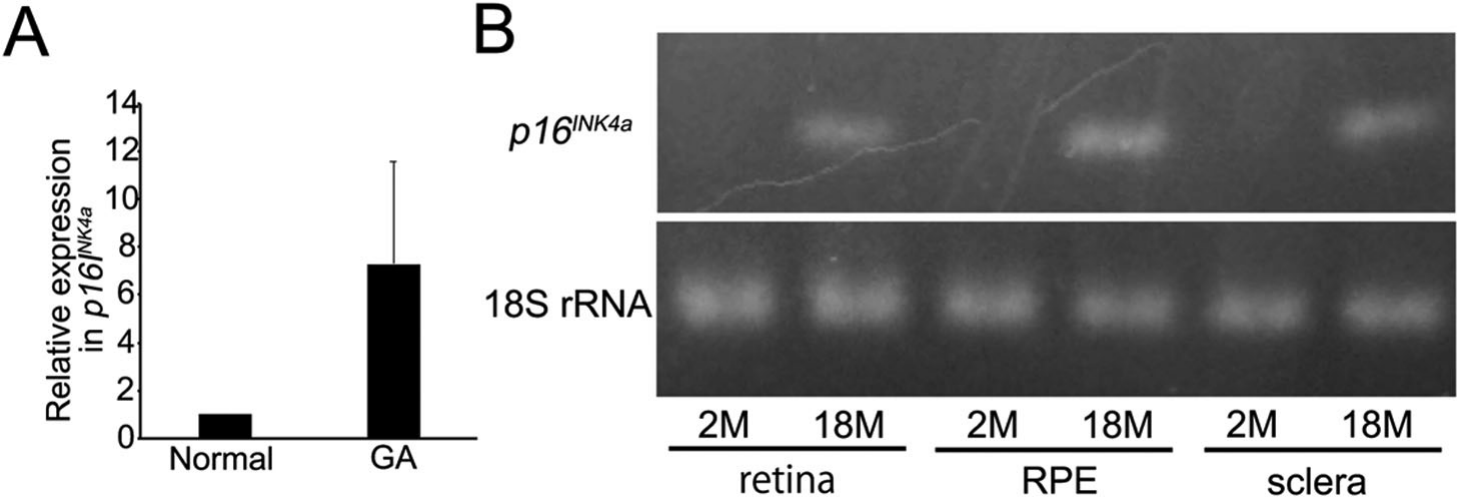
*p16^INK4a^* expression in human and mouse ocular tissues. (**A**) Although the numbers of samples were insufficient for the statistical analysis, *p16^INK4a^* expression in RPE with GA (*n* = 3) (83-year-old female, 92-year-old female, and 91-year-old male) were 10.85-, 8.46-, and 2.55-fold higher, respectively, than that from a normal control subject (*n* = 1) (92-year-old male). (**B**) *p16^INK4a^* expression in 2-month-old and 18-month-old wild-type mice. *p16^INK4a^* was expressed only in 18-month-old mouse eyes.

We examined *p16^INK4a^* expression in human and mouse ocular tissues. We were able to prepare RPE from only three patients with GA and one normal control subject. Although these numbers were insufficient for the statistical analysis, *p16^INK4a^* expression in RPE of patients with GA (83-year-old female, 92-year-old female, and 91-year-old male) was 10.85-, 8.46-, and 2.55-fold higher, respectively, than that in the normal control subject (1.00) (92-year-old male) (Fig. 3). We also examined *p16^INK4a^* expression in 2-month-old and 18-month-old wild-type mice. Because the control sample from the 2-month-old mice did not show abundant *p16^INK4a^* expression, the PCR products were additionally run on a 1.5% agarose gel. *p16^INK4a^* was expressed only in 18-month-old wild-type mouse eyes (Fig. 3). Our results indicate that the RPE from GA-affected eyes and aged mice expressed *p16^INK4a^*.

### Expression of *p16^INK4a^* in Laser-Induced Mouse Subretinal Fibrosis

**Figure 4.**
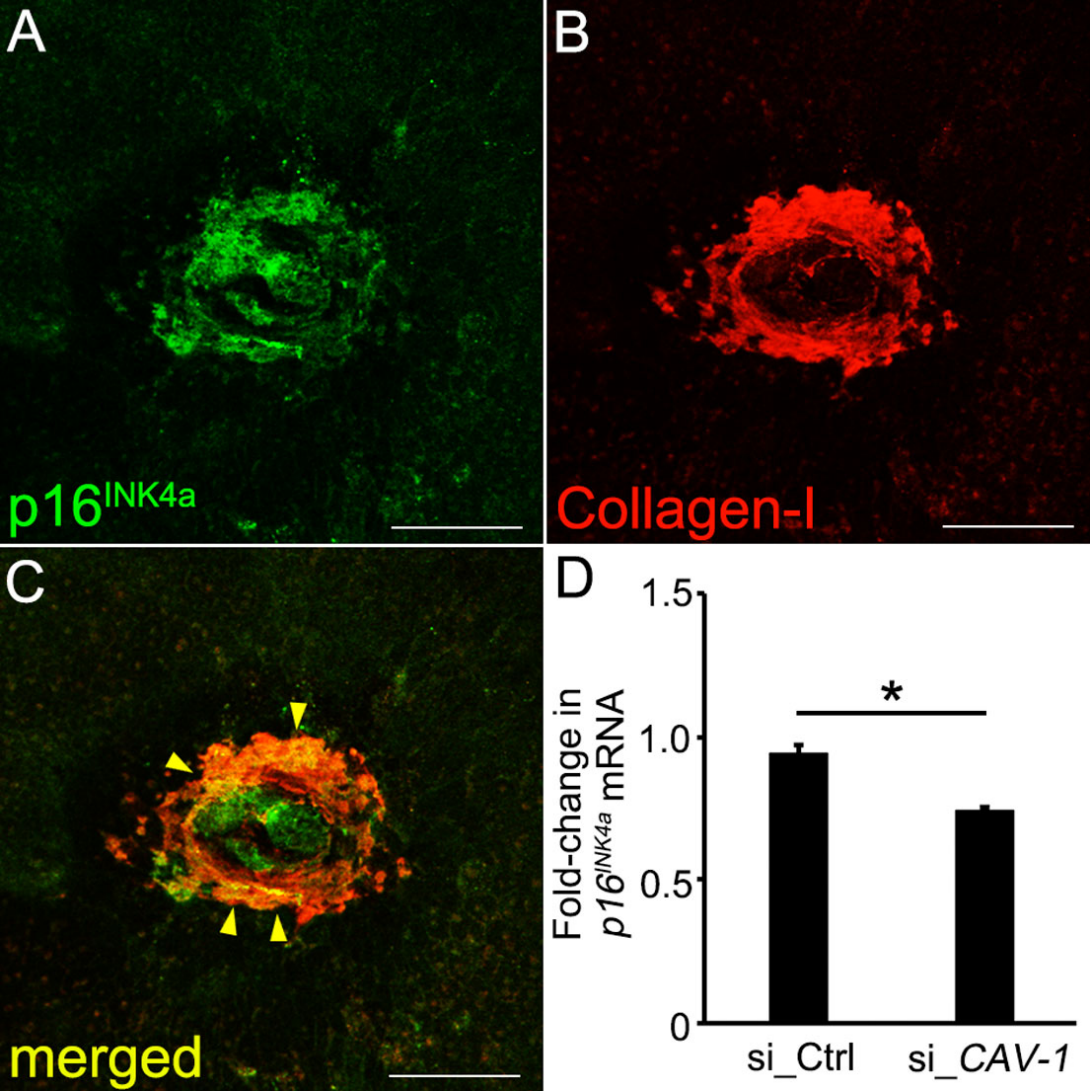
*p16^INK4a^* expression in laser-induced subretinal fibrosis and human RPE cells. (**A**–**C**) RPE/choroid flatmounts stained with (**A**) p16^INK4a^ and (**B**) collagen type I antibodies showed co-localization of p16^INK4a^ and collagen type I in subretinal fibrosis. Eyes were collected at day 35 after laser induction. *Yellow arrowheads* indicate the marginal region of p16INK4a and collagen type I. (**D**) Knockdown of the *CAVEOLIN-1* gene in human RPE cells showed a significant decrease in *p16^INK4a^* expression, *P* = 1.3 × 10^−4^. si_Ctrl = siRNA_control, si_*CAV-1* = siRNA_*CAVEOLIN-1*. Scale bar = 100 µm. ^*^P < 0.05.

Next, we examined *p16^INK4a^* expression in laser-induced subretinal fibrosis. RPE/choroid flatmounts stained with p16^INK4a^ and collaged type I antibodies showed co-localization of p16^INK4a^ and collagen type I in subretinal fibrosis (Fig. 4^5^). Furthermore, we examined caveolin-1–dependent changes of p16^INK4a^ in human RPE cells. The fold-change in *p16^INK4a^* expression in human RPE cells treated with siRNA_*CAVEOLIN-1* was 73.9% ± 1.5% (*n* = 7) compared with the siRNA_control (93.9% ± 3.0%, *n* = 7). Knockdown of the *CAVEOLIN-1* gene in human RPE cells resulted in a significant decrease in *p16^INK4a^* expression, *P* = 1.3 × 10^−4^.

**Figure 5.**
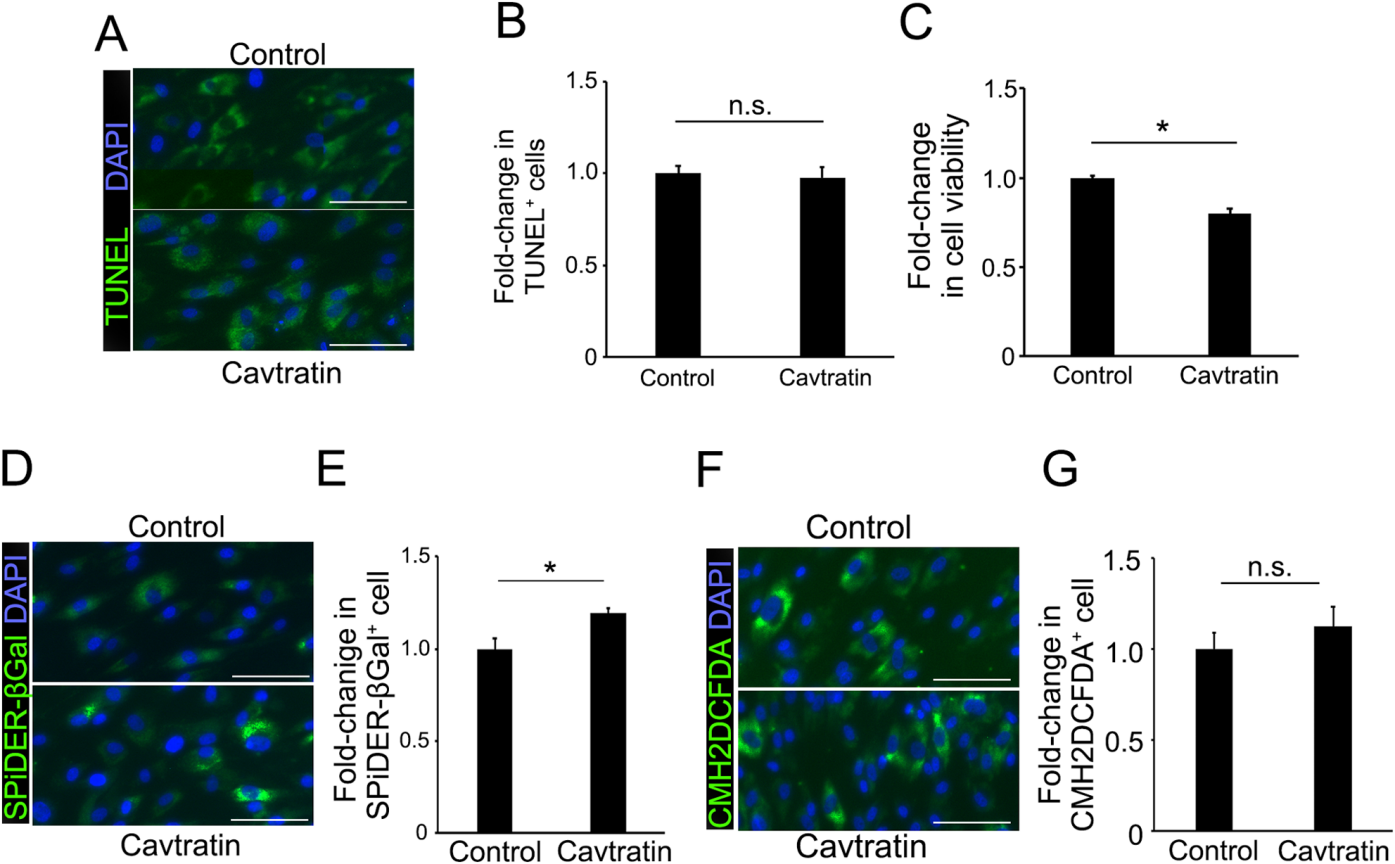
Change in RPE cell fate after cavtratin treatment. (**A**, **B**) TUNEL, an apoptotic cell marker, showed no significant difference between human RPE cells treated with cavtratin and those without treatment (control). (**C**) MTT, a cell viability marker, showed no significant difference between human RPE cells treated with cavtratin control. (**D**, **E**) SPiDER-βGal, a cell senescence marker, showed significant difference between human RPE cells treated with cavtratin and controls. (**F**, **G**) CMH2DCFDA, an reactive oxygen species marker, showed no significant difference between human RPE cells treated with cavtratin and controls. Scale bars = 100 µm, ^*^P < 0.05. n.s. = no significant difference.

### Effect of Cavtratin on RPE Cells

Cavtratin is a cell-permeable peptide of caveolin-1. We examined the effect of cavtratin on cultured human RPE cells by performing a TUNEL (cell apoptosis) assay, cell viability assay, SPiDER-βGal (a senescence cell marker) staining, and reactive oxygen species detection assay (Fig. 5). The percentage of TUNEL-positive cells was 97.8% ± 5.5% (*n* = 10) in RPE cells treated with cavtratin and 100.0% ± 4.2% (*n* = 10) in cells treated with antennapedia (control), with no significant difference, *P* = 0.84. The percentage of cell viability was 80.1% ± 2.9% (*n* = 20) in RPE treated with cavtratin and 100.0% ± 1.4% (*n* = 16) in controls, and the RPE cell viability was significantly reduced by cavtratin, *P* = 3.1 × 10^−6^. The percentage of SPiDER-βGal–positive cells was 119.1% ± 3.1% (*n* = 21) in RPE treated with cavtratin and 100.0% ± 5.9% (*n* = 24) in controls, and the number of SPiDER-βGal–positive senescent cells was significantly increased by cavtratin, *P* = 0.01. The percentage of CMH2DCFDA-positive cells was 112.2% ± 11.0% (*n* = 9) in RPE treated with cavtratin and 100.0% ± 8.5% (*n* = 10) in controls, with no significant difference, *P* = 0.10. Overall, these results suggest that caveolin-1 enhancement did not affect RPE cell apoptosis or reactive oxygen species generation but that it reduced cell viability and increased SPiDER-βGal–positive (senescent) cells.

## Discussion

Caveolin-1 has been a focus of research in ocular science because of its importance in many eye diseases. Caveolin-1 is not expressed in the normal lens epithelium,^19^ but it is upregulated once EMT has been triggered.^12^ Jiang et al.^16^ recently revealed that, in a mouse neovascular AMD model, caveolin-1 was expressed not in the RPE cells but in microglia and macrophages surrounding CNV, and its enhancement contributed to a reduction in CNV growth. The present study showed that in subretinal fibrosis associated with more advanced stages of neovascular AMD, caveolin-1 enhancement effectively decreased fibrosis by blocking EMT in RPE cells. We injected cavtratin only at day 0. Multiple injections might enhance the effect of cavtratin to suppress the subretinal fibrosis. However, a previous study showed that collagen type I–positive subretinal fibrosis was initiated immediately after laser induction^31^ and, therefore, cavtratin injection at day 0 supposedly suppresses the initiation of subretinal fibrosis. By contrast, when we performed a subanalysis of the results of subretinal fibrosis by comparing wild-type and *Cav-1^−/−^* mice, the volume of subretinal fibrosis without cavtratin treatment in *Cav-1^−/−^* mice was significantly larger than that in wild-type mice (Supplementary Figure). Interestingly, even the volume of subretinal fibrosis with cavtratin treatment in *Cav-1^−/−^* mice was significantly greater than that in wild-type mice (Supplementary Figure). These results indicate that cavtratin treatment did not completely cancel the effect of genetic deletion of *Caveolin-1*.

The study by Jiang et al.^16^ and our current study illustrate the therapeutic advantage of caveolin-1 enhancement to control both CNV and subretinal fibrosis. However, we showed that caveolin-1 promotes cellular senescence of RPE cells, which is similar to the change shown in mouse embryonic fibroblasts.^24^ We confirmed increased *p16^INK4a^* expression in the RPE cells from human patients with GA and old mice. In addition, we found that *p16^INK4a^* expression is positively correlated with caveolin-1 expression. It is very interesting that p16 ^INK4a^ and subretinal fibrosis were colocalized in the late-stage mouse AMD model, which indicated that p16^INK4a^ is also expressed during the process of subretinal fibrosis. p16^INK4a^ is known to arrest the cell cycle and, thus, it is also associated with cellular senescence.^32^ Arresting the cell cycle is also associated with EMT suppression. It has been reported that abnormal blood vessels in an animal model of retinopathy of prematurity were positive for senescence-associated β-galactosidase.^33^ A further understanding of the biological relationship between arresting fibrosis and aging is required. In addition, how RPE senescence is involved in the pathogenesis of advanced AMD, including CNV progression and RPE atrophy, remains unclear. However, some studies have suggested that cellular senescence is an important key underlying the mechanism of GA in AMD.^25^^–^^27^ Based on the conclusion from these studies, it is highly possible that RPE senescence promotes RPE atrophy in GA of late-stage AMD.

In summary, enhanced expression of caveolin-1 successfully blocked EMT of the RPE, resulting in the reduction of subretinal fibrosis in neovascular AMD. Nevertheless, in exchange for blocking subretinal fibrosis, caveolin-1 promotes RPE cellular senescence and might ironically promote progression of GA in AMD.

## Supplementary Material

Supplement 1
