# Supplementary material for: Caveolin-1 Promotes Cellular Senescence in Exchange for Blocking Subretinal Fibrosis in Age-Related Macular Degeneration
Source: Invest Ophthalmol Vis Sci. 2020 Sep 14;61(11):21. doi: 10.1167/iovs.61.11.21 (PMC7490224; doi:10.1167/iovs.61.11.21)
Supplement: Supplement 1 [file iovs-61-11-21_s001.pdf]

Supplementary Figure

Caveolin-1 promotes cellular senescence in exchange for blocking subretinal fibrosis in age-related macular degeneration

Hideyuki Shimizu, Kazuhisa Yamada, Ayana Suzumura, Keiko Kataoka, Kei Takayama, Masataka Sugimoto, Hiroko Terasaki, Hiroki Kaneko

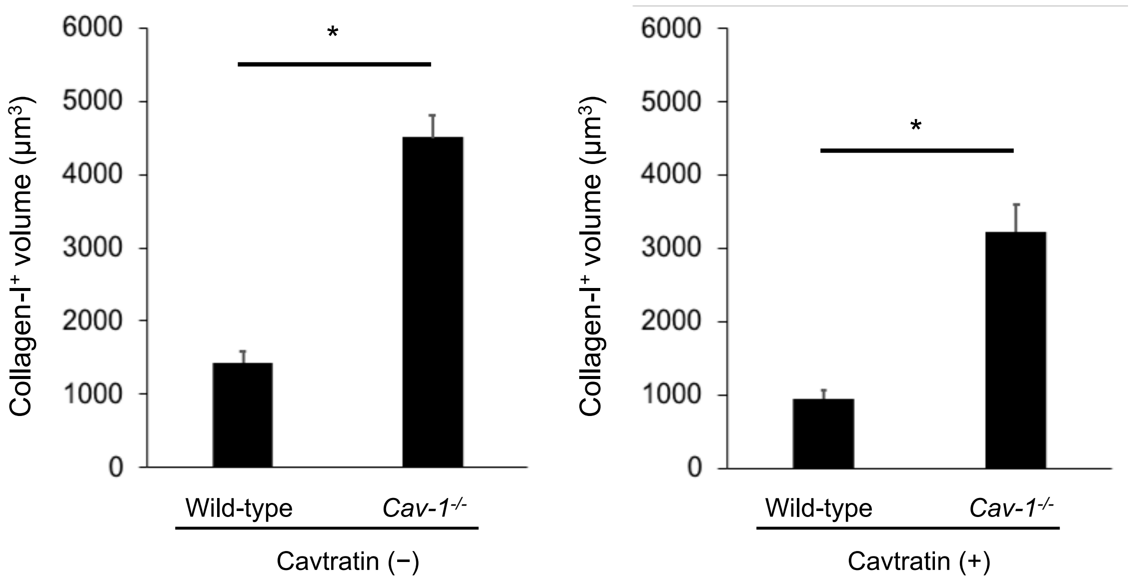

The volume of collagen type I-positive subretinal fibrosis in wild-type mice (1420.4 ± 164.7 μm^3, n = 27) was significantly smaller than that in *Cav-1*$^{-/-}$ mice (4502.8 ± 310.6 μm^3, n = 22, P < 0.001). Similarly, the volume of collagen type I-positive subretinal fibrosis in cavtratin-injected eyes from wild-type mice (950.4 ± 116.2 μm^3, n = 25) was significantly smaller than that in *Cav-1*$^{-/-}$ mice (3232.5 ± 375.5 μm^3, n = 27, P < 0.001).
